# The role of occupational medicine in the response to the coronavirus outbreak: the Tunisian Occupational Health and Safety Institute´s experience

**DOI:** 10.11604/pamj.2022.41.19.27713

**Published:** 2022-01-07

**Authors:** Nesrine Kammoun, Mejda Bani, Habib Nouaigui

**Affiliations:** 1 Institute of Health and Safety at Work, Faculty of Medicine of Tunis, University Tunis El Manar, Tunis, Tunisia

**Keywords:** COVID-19, occupational medicine, disaster planning and response

## Editorial

### Introduction

As COVID-19 spreads globally, companies must plan how to continue their activities and should get involved in the government´s health contingency plan. During this pandemic, workers have been susceptible not only to contracting the disease in the workplace, but also to transmitting it to the community [1]. In this crisis, public health authorities and employers have an important part to play in reducing the impact of the COVID-19 outbreak on businesses, workers and the public. It is therefore the role of occupational and environmental physicians to provide guidance and advice on COVID-19 to companies and help them to mitigate the consequences of the COVID-19 pandemic in the work place [2]. This editorial aims to look at what occupational medicine can add to these concerns and to point up the role of the Tunisian Occupational Health and Safety Institute (TOSHI) during this pandemic.

### Before the lockdown (prior to 22 March 2020)

Since the first case of novel coronavirus was reported in Tunisia in March 2020, the TOSHI has developed guidance for a business intervention plan based on previous outbreak experiences and the government´s contingency plan. The document guided occupational physicians who were advising workplaces on COVID-19 to complement public health measures while reducing disruption to the business. Hence, companies (assisted by their occupational practitioners) could respond effectively to this crisis when cases of infected patients occurred in the company. The TOSHI has also elaborated a business continuity plan for business leaders. The document highlighted specific recommendations and the measures to be taken to maintain the continuity of business activity during the pandemic. Previously, the TOSHI planned for influenza pandemics in the H1N1 pandemic. The novel guide involved updating plans to address the specific exposure risks and other unique characteristics of SARS-CoV-2. The TOSHI recommended that companies implement a dedicated response in line with the public health advice and the business continuity and intervention plan, to mitigate the risk of COVID-19 in workplaces.

#### Communication and information

During this phase, training and information on the responsibilities of each employee in the prevention of disease should be provided by health practitioners. Companies must assume optimal prevention to protect the health of their employees when working, in line with the provisions of article 152 of the Tunisian labor code [3]. Otherwise, workers need information and direction regarding actions taken by their employers to safeguard their health. Considering the current public health advice and the business response plan, the occupational health services are well placed to educate and inform employees. The occupational physician gives people the facts about how the disease is transmitted and how the COVID-19 outbreak could affect the workplace. Communication, workplace policy and the whole process should acknowledge the potential for worker’s mental health and wellbeing to be affected during the pandemic. In this context, the TOSHI under the ministry of health also organized awareness campaigns on COVID-19 at the Assembly of People's Representatives (APR), at the Tunisian Electricity and Gas Company, at the National Social Security Fund (NSSF), at the Office of Civil Aviation and Airports (OCAA) and Tunisian National Television (TNT).

#### Measures to reduce the spread of COVID-19 in workplaces

Again, occupational physicians have particular skills in recognizing exposure to hazards in the workplace and introducing effective measures to reduce the risk to workers´ health. These skills may contribute to mitigating the risk of COVID-19 in workplaces. To assess the risk exposure in the workplace, the occupational physician has to review key exposure factors in the work setting: does the work setting require close contact with people potentially infected with the COVID-19 virus? Do specific job duties require close, repeated, or extended contact with people with known or suspected COVID-19? And has the community spread of the virus included cases in the workplace? When a community outbreak occurs, any workplace or event location where people gather has a high potential for exposure. Healthcare workers have an increased risk of exposure and infection in their workplaces. However, a wide range of service-economy workers may also be at greater risk during pandemics, where transmission is through face-to-face or close contact. Data on the COVID-19 occupational risk, workplace response training and interventional measures may contribute to increased health and safety awareness for responders and workers with potential exposure to COVID-19 and minimize the risks of transmission in the workplace. The workers should comply with four key measures: frequent hand washing, maintaining physical distancing of at least one meter, avoiding touching nose, mouth and eyes, and practicing good respiratory hygiene to cover nose and mouth when coughing or sneezing [4]. These measures are based on the likelihood that the virus is transmitted through large airborne droplets or from the surface and dermal contamination of those droplets. Promoting regular cleaning and disinfection procedures, mainly of frequently touched objects and surfaces, can be helpful to prevent the spread of infections in workplaces [5]. Employers should start practicing these measures even if COVID-19 has yet to reach the communities where they operate. In this context, the TOSHI produced TV spots that were aired on national television to help raise awareness of COVID precautions in workplaces.

### During the lockdown (22 March- 04 May 2020)

The occupational physician is called upon to continue implementing the prevention plan drawn up and to take a collaborative approach with employers and employees and provide advice and support on implementing the required measures in the workplace. Occupational health and safety inspectors carried out inspections of workplaces that remained active during the confinement period. The principal aim of the visits is to look for evidence that measures set out in the business response plan have been implemented in the workplace. They checked safety posters were in place, ensured that hand sanitizing facilities were available at the entrance and exit points to the workplace, that toilets and washing facilities were available and that the one-meter social distancing rule was adhered to along with the route to managing a suspected case. On 02 May 2020, the Tunisian government issued decree n°2020-208 in the Official Gazette setting out the requirements for targeted containment following the requirements of preventing the spread of SARS-CoV-2 [6]. Elderly people, pregnant women and people with chronic conditions (such as diabetes, hypertension, obstructive respiratory disease, heart disease, renal or liver failure, and cancer) - have higher mortality from COVID-19 [7]. Article 10 stated that these would remain subject to the prescriptions of total containment. Article 11 of this decree stipulated that a manual of procedures would be established and published to the public by the TOSHI in order to help companies in the gradual resumption of economic activities [6]. In this context, the TOSHI, in collaboration with the Directorate of Medical Inspection and Occupational Safety (DMIOS), has drafted a generic guide and specific guides to health measures for the prevention of COVID-19 to the oriented resumption of professional activity in the public service, the call centers, the trade sector, the agro-food industry and the public service. Guidance and fact sheets have also been drawn up for hair salons and barbershops, agricultural workers, bakeries and confectionery, small trades, cafes and tea rooms, road transport of goods, retail, and food delivery services [8]. The preferred approaches to control the exposure pathways are to use a combination of strategies to shield workers, including engineering and administrative controls and medical screening of employees. Engineering controls include specialized ventilation to remove hazards from the air, according to the guide to measures for the use of air conditioners in prevention against COVID-19 published by the Ministry of Health. Other structural measures - like simple, clear plastic screens and physical barriers in some customer-facing roles - may protect from COVID-19. Physical distancing is an effective way to help reduce the risk of coronavirus exposure. Through the response plan manual, the TOSHI provided the following guidance to help employers implement social distancing in the workplace: maintain a distance of one meter between each other wherever feasible, including breaks; arrange workplace and common areas to ensure minimal contact between employees; encourage flexible worksites and flexible work hours if feasible; discourage unnecessary gatherings. Enhanced administrative measures might also be needed. Administrative controls involved guidance on a business continuity plan for an outbreak in the workplace, occupational health policies, controlled access to the workplace, cleaning and disinfection procedures for the workplace, and managing the COVID-19 risk when organizing meetings. Medical screenings of employees concerned risk communication among employees, regular follow-up of workers, isolating any worker who began to exhibit symptoms until they could either go home or leave to seek medical care and dealing with sick leave absence according to the advice provided by the national authorities.

### During the targeted de-confinement (04 May - 14 June 2020)

The TOSHI has provided trainees to occupational physicians who are advising workplaces on COVID-19 to tackle the consequences of the novel coronavirus outbreak for some specific sectors. Specialists also provided webinars to advise occupational physicians and hygienists on how to assume their role in the implementation of the guidance and procedure manuals drawn up in the previous phase and the provisions of decree n°2020-208 of the 02 May 2020. Guidance and information related to culture and leisure sectors were also developed in partnership with the Coronavirus committee (the Ministry of Health) to support their planning for service recovery as COVID-19 restrictions were lifted. The set of resources concerned art galleries, museums, archaeological sites, conservatories, movie theatres and festivals. The toolkit was designed to meet the practical needs of the culture and leisure sectors, such as social distancing, behavioral changes, cleaning and hygiene procedures, and communications to staff and users. In addition, the TOSHI worked closely with the government to develop sanitary protocols and in order to prepare for the resumption of many sector activities in Tunisia after the COVID-19 pandemic. These protocols were intended to enable the safe reopening of places of worship, the resumption of tourism and of the fisheries and aquaculture sectors. Other COVID-19 health and safety manuals were approved together with the Ministry of Health and the Ministry of Transport regarding medical tourism, and the management of sites to be used as isolation and quarantine facilities for temporary confinement.

### The TOSHI continued to support the occupational health community

The TOSHI in partnership with the World Health Organization collaborating country office in Tunisia and the National Observatory of New and Emerging Diseases conducted rapid response teams training of trainers workshop for occupational physician in October 2020. The training course aims to ensure a prompt and effective response to public health hazards with a focus on COVID-19. Morever in response to the dynamic circumstances of the public health emergency responses, the TOSHI, in collaboration with the Ministry of Health and the Directorate of Medical Inspection and Occupational Safety, published Interim Guidance for managing workers who have come into close contact with a person with confirmed COVID-19 [9]. The guidance was updated to clarify the strategy for fully vaccinated people. As part of the initiatives to address issues arising from the COVID-19 pandemic, the TOSHI organized several clinical calls to help the occupational health community to tackle COVID-19 vaccine hesitancy and increase uptake by providing information about COVID-19 vaccination. These webinars, held during the year 2021, focused on the following topics: the return to work criteria for employees with SARS-CoV-2 infection, long COVID and workability, guidance for Antigen Testing for SARS-CoV-2 in workplaces, the planning process for hosting a workplace COVID-19 vaccination program and how to set up vaccination on-site at the workplace.

## Conclusion

The COVID-19 crisis has shed a different light on the critical dimension of the TOSHI´s role, which is to provide guidance and support the continuity of business during the COVID-19 pandemic. Occupational medicine is still an integral part of the national management of the COVID-19 outbreak. This role depends on a long history of occupational physician’s involvement as both clinicians and public health officers engaged in protecting the national workforce from the economic and health consequences of a pandemic and other disasters.
